# Bonding Properties between Fly Ash/Slag-Based Engineering Geopolymer Composites and Concrete

**DOI:** 10.3390/ma16124232

**Published:** 2023-06-07

**Authors:** Baogui Wang, Hu Feng, Hao Huang, Aofei Guo, Yiming Zheng, Yang Wang

**Affiliations:** 1 Yellow River Laboratory, Zhengzhou University, Zhengzhou 450001, China; 2 Zhengzhou Metro Group Co., Ltd., Zhengzhou 450000, China; 3 China Institute of Water Resources and Hydropower Research, Beijing 100038, China; 4 School of Water Conservancy and Civil Engineering, Zhengzhou University, Zhengzhou 450001, China

**Keywords:** engineering geopolymer composite (EGC), PVA fiber, PE fiber, tensile property, bond performance

## Abstract

Concrete infrastructure repair remains a formidable challenge. The application of engineering geopolymer composites (EGCs) as a repair material in the field of rapid structural repair can ensure the safety of structural facilities and prolong their service life. However, the interfacial bonding performance of existing concrete with EGCs is still unclear. The purpose of this paper is to explore a kind of EGC with good mechanical properties, and to evaluate the bonding performance of EGCs with existing concrete using a tensile bonding test and single shear bonding test. At the same time, X-ray diffraction (XRD) and Scanning electron microscopy (SEM) were adopted to study the microstructure. The results showed that the bond strength increased with the increase in interface roughness. For polyvinyl alcohol (PVA)-fiber-reinforced EGCs, the bond strength increased with the increase in FA content (0–40%). However, with the change of FA content (20–60%), the bond strength of polyethylene (PE) fiber-reinforced EGCs have little change. The bond strength of PVA-fiber-reinforced EGCs increased with the increase in water–binder ratio (0.30–0.34), while that of PE-fiber-reinforced EGCs decreased. The bond–slip model of EGCs with existing concrete was established based on the test results. XRD studies showed that when the FA content was 20–40%, the content of C-S-H gels was high and the reaction was sufficient. SEM studies showed that when the FA content was 20%, the PE fiber–matrix bonding was weakened to a certain extent, so the ductility of EGC was improved. Besides, with the increase in the water–binder ratio (0.30–0.34), the reaction products of the PE-fiber-reinforced EGC matrix gradually decreased.

## 1. Introduction

Engineered cementitious composite (ECC) is a cement-based composite using cement, sand, and mineral mixture as matrix and a small amount of fibers as reinforcing materials. In general, the ductility of ECC is 500–700 times that of Portland cement concrete (PCC) [1], with an ultimate tensile strain of up to 6% [2,3]. Typical ECC mixtures usually require 2–3 times more cement than PCC mixtures. As the production of ordinary Portland cement is a process with high carbon dioxide emissions, using geopolymers to produce ECC is an effective alternative. Some studies have shown that geopolymers can reduce energy consumption by about 60% and carbon dioxide emissions by about 80% compared with Portland cement production [4,5,6,7,8,9,10,11]. Geopolymer-based ECC produced by completely replacing Portland cement with geopolymer is called engineering geopolymer composite (EGC), and has characteristics of strain hardening and multiple cracking [12].

Fiber-reinforced cement-based materials are currently recognized as effective repair materials to improve mechanical properties and durability of existing concrete. However, fiber-reinforced ordinary Portland cement-based repair materials have a long setting time and slow strength development [13,14], and are not ideal for rapid repair. EGCs have great potential in the field of rapid repair due to their fast hardening, high early strength, and good durability. Therefore, it is necessary to develop an EGC material with excellent mechanical properties and high bonding strength with existing concrete for rapid concrete repair.

There have been some studies on fly-ash-based EGCs. Shaikh [15] studied the mechanical properties of a fly-ash-based EGC reinforced with polyvinyl alcohol (PVA) fibers. The test results showed that the deflection and toughness of the EGC mixes under bending load were slightly better than those of ECC mixes. However, due to the low reactivity of fly ash, the compressive strength of the EGC was relatively low, and the 28-day compressive strength was 17.4–27.6 MPa. Ohno et al. [12] cured the fly-ash-based EGC mixes in the oven at 60 °C, and the 28-day compressive strength, 28-day ultimate tensile stress, and 28-day ultimate tensile strain of the samples were increased to attain up to 27.3 MPa, 3.39 MPa, and 4.26%, respectively. To further improve the mechanical properties of fly-ash-based EGCs, Ohno [16] proposed a new design method integrating experimental design, micromechanics modeling, and material sustainability index. The 28-day compressive strength and ultimate tensile strain of the EGCs reached 43.1 MPa and 4.7%, respectively. At the same time, the energy consumption and CO_2_ emissions decreased by 11.2% and 55.6%, respectively. It can be seen that the ultimate tensile strain of fly-ash-based EGCs can reach 4–5%, but its low strength and high-temperature curing conditions limit its application [17]. 

In recent years, using ground granulated blast furnace slag (GGBS) to replace part of fly ash has become an effective method to improve the performance of fly-ash-based geopolymers and eliminate the necessity of high-temperature curing [18]. The main hydration products produced by GGBS participating in the activated process of fly ash are calcium aluminosilicate hydrate (C-A-S-H) and aluminosilicate-hydrate (N-A-S-H) gels [19,20,21]. Nematollahi et al. [22] studied the mechanical performances of EGCs prepared with fly ash, GGBS, and burnt lime as cementitious materials. The results showed that the 28-day compressive strength of EGC mixes cured in a standard environment was close to that of EGC mixes cured at high temperatures. Furthermore, the 28-day compressive strength, ultimate tensile stress, and ultimate tensile strain of EGC cured at high temperature using a combination of GGBS and low-calcium fly ash as the cementing material were 48.7 MPa, 4.6 MPa, and 4.2%, respectively [23]. Nath and Kumar studied the mechanical performances of geopolymers made of different kinds of fly ash and GGBS. The test results showed that the strength improvement of the mixes was mainly due to the more drastic GGBS reaction and development of a more compact microstructure compared with the one-component geopolymer material made of fly ash [24,25]. Behzad studied the effect of curing conditions on the uniaxial tensile properties of fly-ash- and slag-based EGCs. The research results showed that the ultimate tensile strain of the PVA-fiber-reinforced EGCs cured at room temperature was higher than that cured at high temperature. This is because the room-temperature curing increases the fracture toughness and the chemical bond of the EGCs [23]. 

The bond performance between geopolymer and ordinary concrete is a key indicator of repair quality [26,27]. In recent years, several researchers have considered geopolymer mortar as a repair material for strengthening concrete and have conducted studies on the properties of geopolymers. Alanazi et al. [28] studied the bond strength of the interface between geopolymer and cement mortar through slant shear tests. The research results showed that the bond strengths after 3 days curing through splitting and slant shear tests reached 3.63 MPa and 16.32 MPa, respectively. Additionally, it was found that the failure of the samples mainly occurred in the mortar, which indicated that an excellent bond interface was formed. Zhang et al. [29] studied the tensile bonding properties of fly ash and metakaolin-based geopolymer mortar with ordinary concrete. The research results showed that the tensile bond strength was 2.75 MPa, which was higher than that of ordinary cement with PCC (1.7 MPa). The incorporation of chopped carbon fiber improves the tensile bond strength between geopolymer mortar and ordinary concrete to a certain extent [29]. Tan et al. [30] studied geopolymer mortar with alkali-activated slag cement as cementitious material, sodium silicate as alkali activator, and 0.1% polypropylene fiber and 0.1% basalt fiber as reinforcement materials, and examined its interfacial flexural–tensile strength with ordinary concrete. The results showed that the interfacial flexural–tensile strength of geopolymer mortar was 6.14 MPa, which was higher than that of ordinary cement mortar (5.41 MPa). From the above studies, it can be concluded that geopolymer has good bond performance with concrete and is an ideal repair material. In contrast, practical applications of geopolymer mortar in engineering have revealed that it presents the disadvantages of high brittleness and low anti-deformation ability [31]. The toughness, deformation resistance, and durability of geopolymer mortar can be improved by adding high-performance fibers and nanomaterials. The EGCs prepared by adding high-performance fibers effectively improved the toughness, deformation resistance and durability of geopolymer mortars. Therefore, this study aims to explore the interfacial bonding properties between PVA- and PE-fiber-reinforced EGCs and existing concrete to obtain new repair composites.

The purpose of this research was to explore the possibility of using these EGCs as a repair material. Therefore, this study explores a kind of EGC with good mechanical properties. The bonding properties of EGCs and existing concrete were evaluated using tensile bonding test and single shear bonding test, and the bond–slip model of EGCs and existing concrete was established according to the test results. At the same time, the microstructure characteristics of EGCs were analyzed by X-ray diffraction (XRD) and scanning electron microscopy (SEM) results. The innovation of this study is to explore the possibility of EGC as a repair material through bonding tests. At the same time, it provides some theoretical support for the application of EGCs in the field of civil engineering.

## 2. Materials and Experiments

### 2.1. Materials

**Figure 1:**
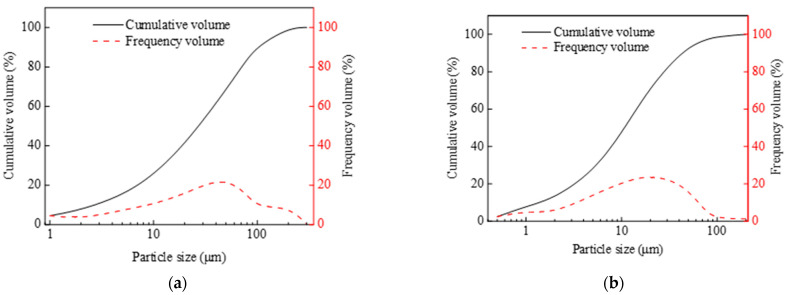
Particle size distribution: (**a**) FA and (**b**) GGBS.

**Figure 2:**
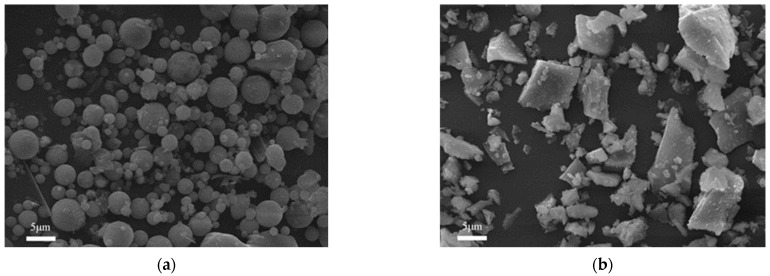
SEM images of the FA and GGBS: (**a**) FA and (**b**) GGBS.

Sodium hydroxide (NaOH, 99 wt.%) and sodium silicate (Na_2_SiO_3_) solution (SiO_2_ = 27.7%, Na_2_O = 8.8%, and water = 63.5%, supplied by Changlong Alkali Factory, Gongyi, China) were mixed at a mass ratio of 0.15 to prepare alkali activator solution with a SiO_2_/Na_2_O molar ratio of 1.4. FA and GGBS were used as cementitious materials. The FA used in this study was class F with a specific surface area of 357.0 m^2^/kg and density of 2.6 g/cm^3^. The GGBS used in this study was class S95 with a specific surface area of 610.6 m^2^/kg and density of 2.9 g/cm^3^. The chemical compositions of the GGBS, as measured in an X-ray fluorescence (XRF) analysis, are shown in Table 1. The particle size distribution of FA and GGBS obtained by laser particle analyzer (Type: Mastersizer 3000; Malvern Panalytical Company, Malvern, UK) is shown in Figure 1. SEM images of FA and GGBS are shown in Figure 2. PE and PVA fibers were used as reinforcement materials in the mixtures. The properties of PVA and PE fibers are listed in Table 2. In addition, the ordinary Portland cement (P.O.42.5), river sand (fine aggregate) with fineness modulus of 2.88, and crushed limestone (coarse aggregate) with a diameter of 5–20 mm were used to produce existing concrete with a strength grade of C30.

### 2.2. Mix Proportions

Table 3 shows the mix proportions of FA/GGBS-based engineered geopolymer composite (EGC) mixes. The alkali activator solution was prepared by mixing solid NaOH and Na_2_SiO_3_ solutions and adjusting their module. The mass ratio of activator to binder (FA + GGBS) was 4%. The mass ratios of FA to binder were 0%, 20%, 40%, and 60%. The mass ratios of water to binder were 0.30, 0.32, and 0.34. PVA or PE fibers with volume content of 2% were added to the mixture.

### 2.3. Mixing and Sample Preparation

The preparation process of the alkali activator solution was as follows. A certain amount of solid NaOH was weighed, dissolved in water, and mixed with sodium silicate solution. The alkali activator solution was prepared by adjusting the module of sodium silicate solution, and was placed at room temperature for 24 h to achieve chemical equilibrium.

The mixing process was as follows. The FA and GGBS were mixed in proportion and stirred with high-power electric stirrer for 1 min. Then, the alkali activator solution was gradually poured into the blending machine and mixed for 3 min. When the mixture had a certain fluidity and cohesiveness, the PVA or PE fibers were slowly added. The whole stirring process lasted about 7 min. Then, the obtained EGC slurry was cast into the mold and placed on the vibration table for vibration compaction for 2 min.

The prepared EGCs were demolded after curing in the indoor environment for one day. After that, the samples were cured in the curing room (20 ± 1 °C, relative humidity: 90% ± 5% RH) to the age of 8 and 28 days.

### 2.4. Experiments

#### 2.4.1. Compressive Test

The compression test was carried out according to ASTM C109 [32]. For each mix proportion, 3 cubic samples with dimensions of 50 mm × 50 mm × 50 mm were used in the test. The compressive strength of samples was calculated according to Equation (1):

(1) $$f_{m}=\frac{P}{A}$$

where *f_m_* is the compressive strength; *P* is the ultimate load; and *A* is the area.

#### 2.4.2. Uniaxial Tensile Test

**Figure 3:**
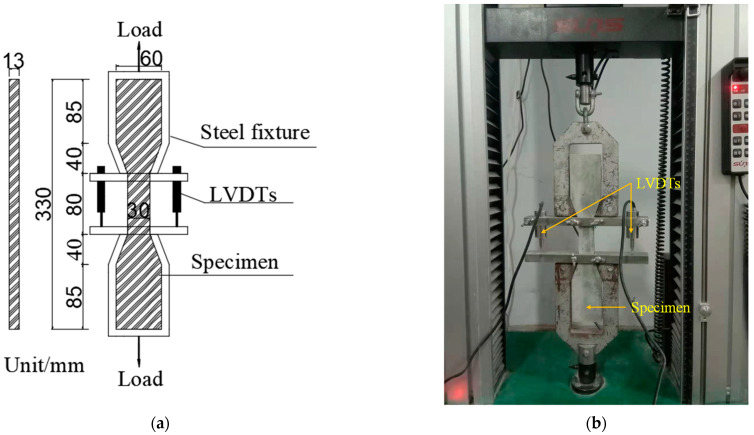
Uniaxial tensile test: (**a**) size of the dog-bone sample and (**b**) test setup.

Dog-bone samples were used for uniaxial tensile tests with reference to the recommendations of the Japan Society of Civil Engineers [33]. A servo hydraulic universal testing machine (maximum load capacity: 2 kN Type: UTM6203, Suns Technology Company, Shenzhen, China) was used for the test. Figure 3 shows the dog-bone sample size and test setup. During the test, the dog-bone sample bore the tensile load, and the displacement rate was adjusted to 0.20 mm/min. Two linear variable differential sensors (LVDTs) with a range of 10 mm were installed in the tensile section of the tensile samples to monitor the displacement. The load and displacement data of the dog-bone samples were recorded by the computer. The tensile stress was obtained by dividing the tensile load by the cross-sectional area of the dog-bone sample tensile section, and the tensile strain was obtained by dividing the tensile displacement by the gauge length (80 mm), so as to draw the stress–strain curve. The tensile stress and tensile strain can be calculated by Equations (2) and (3), respectively. The maximum tensile stress of dog-bone sample and its corresponding tensile strain were denoted as the ultimate tensile stress and ultimate tensile strain, respectively.

(2) $$\sigma =\frac{F}{bh}$$

where *σ* is the tensile stress; *b* is the width of a dog-bone sample; *F* is the tensile load; *h* is the thickness of a dog-bone sample.

(3) $$\varepsilon =\frac{L_{0}}{L}$$

where *ε* is the tensile strain; *L*_0_ is the tensile displacement; *L* is the gauge length.

#### 2.4.3. Tensile Bonding Test

**Figure 4:**
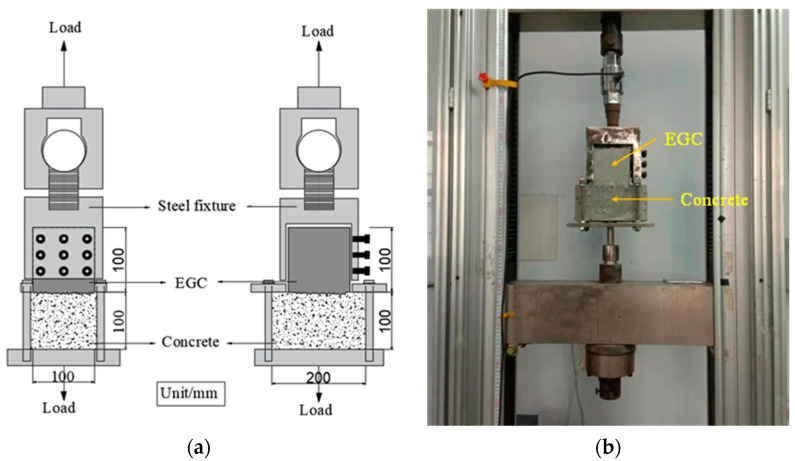
Tensile bonding test: (**a**) schematic illustration and (**b**) test setup.

The tensile bond strength of the interface between EGC and existing ordinary concrete was tested using the tensile bonding test, as shown in Figure 4. A strength grade C30 concrete block with a size of 100 × 100 × 200 mm^3^ was cast. After standard curing for 28 days, the interface was treated with a high-pressure water jet and a chiseling machine. The interface roughness was measured using the sand-filling method [34]. The specific operation is as follows. The treated bonding surface was blown with an air compressor, washed with water, and dried. The sand was poured onto the bonding surface and troweled with a scraper. Then, the sand was poured out to measure its volume. The average height was obtained by dividing the sand volume by the bonding surface area, and was used as the interface roughness. Then, the 100 mm × 100 mm × 100 mm EGC was cast along the horizontal direction of the concrete block and cured for 28 days. The bond area of the concrete with the EGC was 100 mm × 100 mm. The universal testing machine was used for the loading test, and the loading speed was set to 0.2 mm/min. The tensile bond strength can be calculated by Equation (4):

(4) $$f_{t}=\frac{F}{A}$$

where *f_t_* is the tensile bond strength; *A* is the bond area; *F* is the ultimate tensile load.

#### 2.4.4. Single Shear Bonding Test

**Figure 5:**
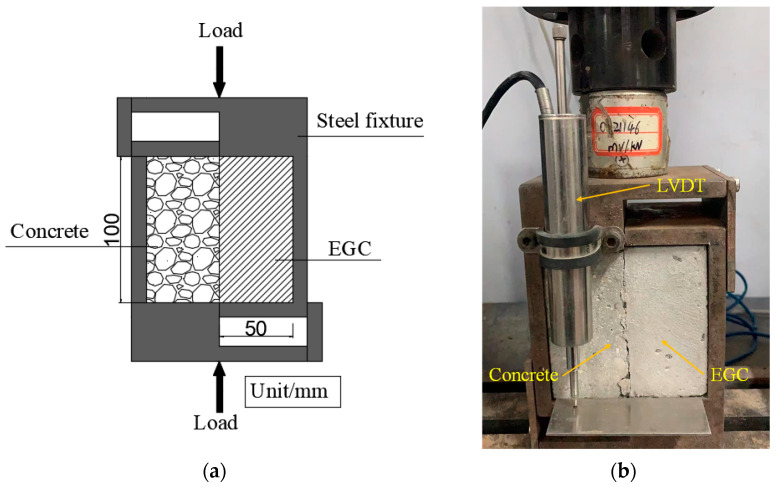
Single shear bonding test: (**a**) schematic illustration and (**b**) test setup.

The shear resistance of the interface between existing concrete and EGC was studied using the single shear bonding test, as shown in Figure 5. The 50 mm × 100 mm × 100 mm C30 concrete block was cast in advance, on which the interface roughness treatment (high-pressure water jet and chiseling machine) was carried out after curing for 28 days. Then, the 50 mm × 100 mm × 100 mm EGC sample was cast and cured for 28 days to form a cube sample of 100 mm × 100 mm × 100 mm for use in the single shear bonding test. A servo hydraulic universal testing machine (Type: WDW-100, maximum; load capacity: 100 kN; manufacturer: Suns Technology Company, Shenzhen, China) was used for the test. The displacement rate was controlled at 0.25 mm/min. The maximum shear load and relative slip of samples were recorded, based on which the shear stress–interface slip curve was drawn. The ultimate shear strength was calculated according to Equation (5):

(5) $$\tau_{u}=\frac{P_{u}}{A}$$

where *τ_u_* is the ultimate shear strength; *P_u_* is the maximum shear load; and *A* is the bond area.

#### 2.4.5. X-ray Diffraction (XRD) Test

The chemical composition of geopolymer hydration products was analyzed by using the X ‘Pert3 powder diffractometer produced by PANalytical Company, Malvern, United Kingdom. The geopolymer samples were first soaked in ethanol for 2 days to terminate hydration and then were dried in a 50 °C oven for 2 days. The particles passing the 0.075 mm sieve were collected for analysis. The diffraction patterns were collected when the 2θ angle was between 5° and 80°, with a 0.02° step size. The scanning speed was 10°–20°/min.

#### 2.4.6. Scanning Electron Microscopy (SEM) Test

ZEISS EVO 10 (Carl Zeiss, Jena, Germany) scanning electron microscope was used to analyze the microstructure of PE-fiber-reinforced EGCs. Before testing, the tensile section of the dog-bone samples was cut into blocks of 1 × 1 × 0.3 cm^3^ and placed in anhydrous ethanol for 2 days to terminate hydration. After that, the samples were dried at 50 °C in a vacuum drying oven. The surfaces of the samples were sprayed with gold to make them have excellent electrical conductivity.

## 3. Results and Discussions

### 3.1. Compressive Strength

#### 3.1.1. Influence of FA Content

**Figure 6:**
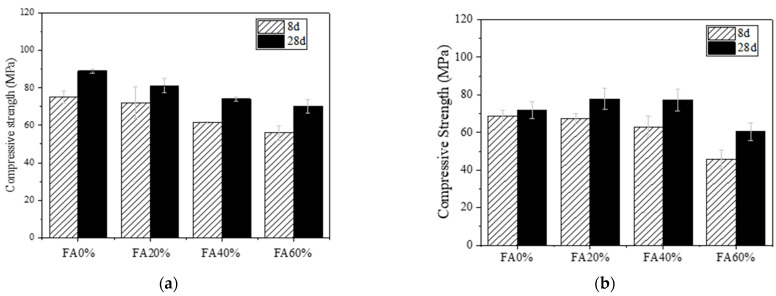
Compressive strength of EGCs with different FA content: (**a**) W0.32-PVA and (**b**) W0.32-PE.

Figure 6 shows the compressive strength results of PVA and PE-fiber-reinforced EGCs with different FA contents. It can be seen from Figure 6a that, for PVA-fiber-reinforced EGC, with the increase in FA content, the compressive strength at the age of 8 and 28 days decreases, and the mix with 0% FA has the highest compressive strength at 75 MPa and 89 MPa, respectively. It can be seen from Figure 6b that, for PE-fiber-reinforced EGC, with the increase in FA content, the compressive strength at the age of 8 days decreases. Additionally, with the increase in FA content, the compressive strength of the sample at the age of 28 days first increases and then decreases. The mix with 20% FA has the highest 28-day compressive strength of 79 MPa, which may be due to the best formation of aluminosilicate hydrate and calcium silicate hydrate (C-S-H) gels [35]. The decrease in compressive strength caused by 60% FA may be due to the high content of FA reducing the reaction activity [36]. Meanwhile, it can be seen from Figure 6 that the compressive strength of PVA-fiber-reinforced EGCs with the same FA content is higher than that of PE-fiber-reinforced EGCs. This could be due to the lower aspect ratio of the PVA fibers than the PE fibers, which suggests that the PVA fibers may induce less fiber damage (air entrapping effect) in the composites compared to the PE fibers [37].

The compressive strengths of the PVA-fiber-reinforced EGCs with FA0%, FA20%, FA40%, and FA60% at the age of 8 days reached 86%, 89%, 83%, and 80% at the age of 28 days, respectively. Additionally, the compressive strengths of the PE-fiber-reinforced EGCs with FA0%, FA20%, FA40%, and FA60% at the age of 8 days reached 95%, 86%, 80%, and 75% at the age of 28 days, respectively. This shows that, for PVA and PE-fiber-reinforced EGCs, the mix with high GGBS content has high early strength and rapid strength development, which is suitable for rapid repair.

#### 3.1.2. Influence of Water–Binder Ratio

**Figure 7:**
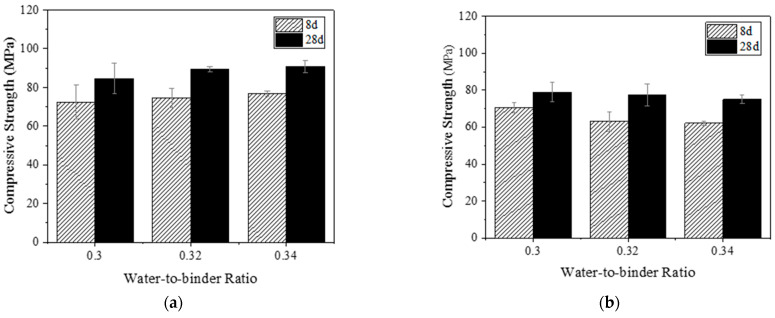
Compressive strength of EGCs with different water–binder ratios: (**a**) FA0%-PVA and (**b**) FA40%-PE.

FA0%-PVA and FA40%-PE samples were selected to analyze the influence of water–binder ratio. Figure 7 shows the compressive strength results of PVA and PE-fiber-reinforced EGC with different water–binder ratios. As shown in Figure 7a, for PVA-fiber-reinforced EGC, with the increase in water–binder ratio, the compressive strengths at 8 and 28 days show an upward trend. Compared to the sample with a water–binder ratio of 0.30, the 8-day compressive strengths of the samples with water–binder ratios of 0.32 and 0.34 increased by 3% and 6%, respectively; the 28-day compressive strengths of the samples with water–binder ratios of 0.32 and 0.34 increased by 6% and 7%, respectively. This phenomenon may be due to the fact that the cementitious material is GGBS in the reaction system for FA0%-PVA, and when the water–binder ratio is 0.30, the initial reaction is violent and the fluidity is poor [38], so some GGBS is wrapped by the hydration products and cannot participate in the reaction, resulting in insufficient reaction and thus low compressive strength. With the increase in water–binder ratio, the fluidity of the EGC slurry is improved, and more GGBS participates in the hydration reaction, resulting in higher compressive strength.

As shown in Figure 7b, for PE-fiber-reinforced EGC, with the increase in water–binder ratio, the compressive strengths of the sample at the age of 8 and 28 days show a downward trend but within a limited range. Compared to the sample with the water–binder ratio of 0.30, the 8-day compressive strengths of the samples with the water–binder ratios of 0.32 and 0.34 were reduced by 11% and 12%, respectively; the 28-day compressive strengths of the samples with the water–binder ratios of 0.32 and 0.34 were reduced by 2% and 5%, respectively. This shows that a water–binder ratio in the range of 0.30–0.34 has little effect on the 28-day compressive strength of PE-fiber-reinforced EGCs.

### 3.2. Uniaxial Tensile Property

#### 3.2.1. Influence of FA Content

**Figure 8:**
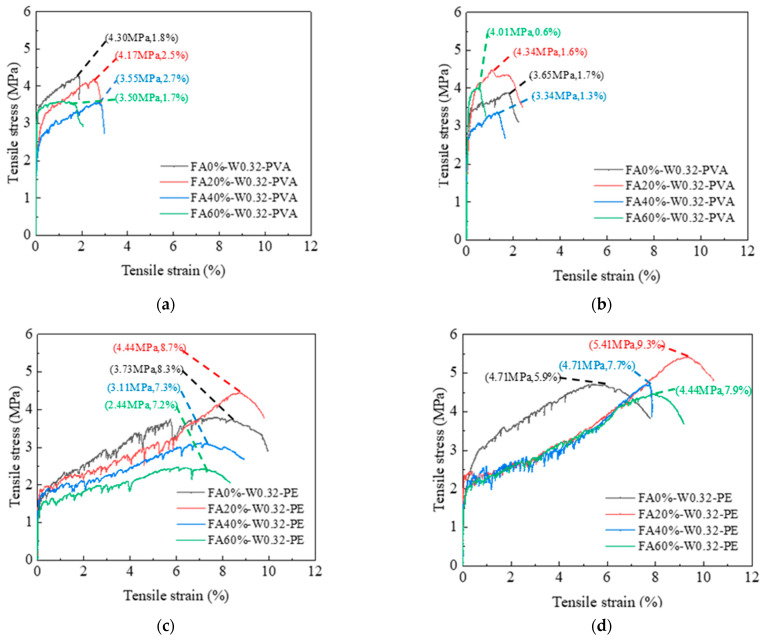
Tensile stress–strain curves of EGCs with different FA contents: (**a**) 8d-PVA, (**b**) 28d-PVA, (**c**) 8d-PE, and (**d**) 28d-PE.

Figure 8 shows the tensile stress–strain curves of PVA- and PE-fiber-reinforced EGC with different FA contents. Figure 8a shows that, for PVA-fiber-reinforced EGC, when the FA contents are 0%, 20%, 40%, and 60%, the ultimate tensile strains of samples at 8 days were 1.8%, 2.5%, 2.7%, and 1.7%, respectively, and the ultimate tensile stresses were 4.30 MPa, 4.17 MPa, 3.55 MPa, and 3.50 MPa, respectively. The ultimate tensile strain first increased and then decreased with the increase in FA content, and the ultimate tensile stress decreased with the increase in FA content. Figure 8b shows that the ultimate tensile strains of the PVA-fiber-reinforced EGC samples at 28 days were 1.7%, 1.6%, 1.3%, and 0.6%, respectively, and the ultimate tensile stresses were 3.65 MPa, 4.34 MPa, 3.34 MPa, and 4.01 MPa, respectively. The ultimate tensile strain decreased with the increase in FA content, but the ultimate tensile stress showed no obvious regularity. The reason for the decrease in ultimate tensile strain is that with the increase in FA content, the strengths of the chemical bond and friction bond between the geopolymer matrix and PVA fiber became higher [35]. When the tensile stress exceeded the ultimate tensile strength of the matrix, the PVA fiber is not easily pulled out, and breaks, resulting in the reduction in the ultimate tensile strain.

Figure 8c shows that, for PE-fiber-reinforced EGC cured for 8 days, compared to FA20%, the ultimate tensile stresses of FA0%, FA40%, and FA60% were reduced by 16%, 30%, and 45%, respectively, and the ultimate tensile strains were reduced by 5%, 16%, and 17%, respectively. Figure 8d shows that, for the PE-fiber-reinforced EGC samples cured for 28 days, compared to FA20%, the ultimate tensile stresses of FA0%, FA40%, and FA60% were reduced by 13%, 13%, and 18%, respectively, and the ultimate tensile strains were reduced by 37%, 17%, and 15%, respectively. The results show that, with the increase in FA content, the ultimate tensile stress and ultimate tensile strain of the sample first increased and then decreased, and the tensile property of the FA20% sample was the best. As mentioned previously, the enhanced ultimate tensile stress is a synergistic result of the optimum content of GGBS and FA in the EGCs system. CaO in GGBS can participate in FA activation process and produce C-S-H/C-A-S-H gel. The bonding between geopolymer matrix and PE fibers is strengthened [20].

#### 3.2.2. Influence of Water–Binder Ratio

**Figure 9:**
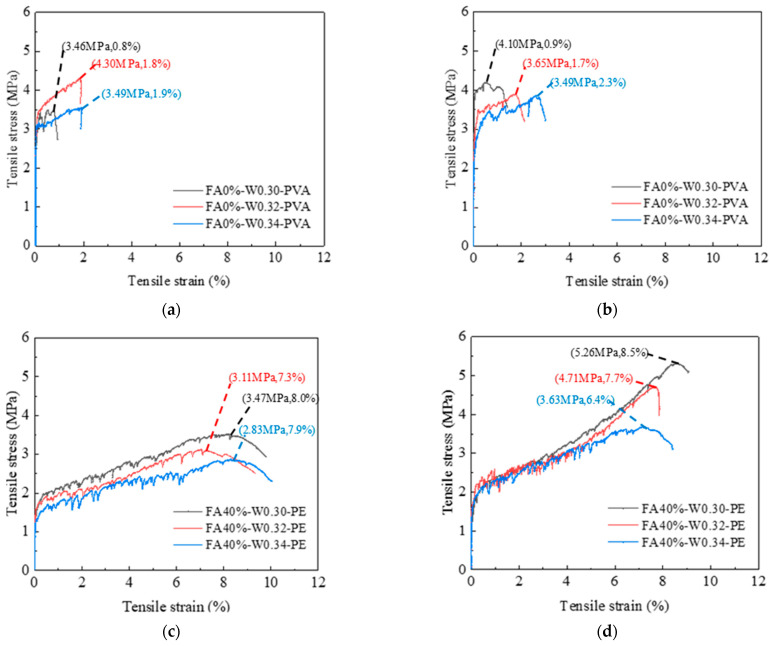
Tensile stress–strain curves of EGCs with different water–binder ratios: (**a**) 8d-PVA, (**b**) 28d-PVA, (**c**) 8d-PE, and (**d**) 28d-PE.

FA0%-PVA and FA40%-PE samples were selected to analyze the influence of water–binder ratio. Figure 9 shows the tensile stress–strain curves of PVA and PE-fiber-reinforced EGCs with different water–binder ratios. Figure 9a shows that, for PVA-fiber-reinforced EGCs at the age of 8 days, compared to samples with a water–binder ratio of 0.30, the ultimate tensile stresses of samples with water–binder ratios of 0.32 and 0.34 increased by 24.3% and 1%, respectively, and the ultimate tensile strains increased by 135% and 141%, respectively. Figure 9b shows that, compared to the samples with a water–binder ratio of 0.30 at the age of 28 days, the ultimate tensile stresses of the samples with water–binder ratios of 0.32 and 0.34 decreased by 12.2% and 14.8%, respectively, while the ultimate tensile strains increased by 92.2% and 156%, respectively. The reason for the improvement of the ultimate tensile strain of the samples with the increase in water–binder ratio may be that the increase in water amount can reduce the friction resistance between the EGC matrix and fibers, leading the fibers to be pulled out more easily rather than broken during the tensile process.

Figure 9c shows that, for PE-fiber-reinforced EGCs at the age of 8 days, compared to the samples with a water–binder ratio of 0.30, the ultimate tensile stresses of samples with water–binder ratios of 0.32 and 0.34 decreased by 11.7% and 18.3%, respectively, and the ultimate tensile strains decreased by 9.9% and 1.6%, respectively. Figure 9d shows that, compared to the samples with a water–binder ratio of 0.30 at the age of 28 days, the ultimate tensile stresses of samples with water–binder ratios of 0.32 and 0.34 were reduced by 11.7% and 30.9%, respectively, and the ultimate tensile strains were reduced by 10.5% and 17.4%, respectively.

In summary, for PVA-fiber- or PE-fiber-reinforced EGCs, the increase in water–binder ratio leads to the decrease in ultimate tensile stress of the sample. This is because when the water content in the matrix is too high, the residual unreacted water in the matrix results in a increase in matrix porosity and thus a decrease in ultimate tensile stress.

#### 3.2.3. Toughening Effect of PVA and PE Fibers

Comparing Figure 8b,d with Figure 9b,d, it is found that the ultimate tensile strain of PE-fiber-reinforced EGCs is significantly higher than that of PVA-fiber-reinforced EGCs. This shows that the toughening effect of PE fiber is significantly better than that of PVA fiber for EGCs. The reason for this phenomenon may be that compared to PE fiber, the tensile strength of PVA fiber is lower. When the EGC cracking strength is high, PVA fiber cannot withstand the large tensile force during cracking, resulting in fiber fracture [39].

### 3.3. Tensile Bond Strength

According to the previous exploration, FA0%-W0.34-PVA and FA40%-W0.32-PE were selected as reference groups to study the influences of interface treatment method, FA content, and water–binder ratio on tensile bond strength due to their better mechanical properties.

#### 3.3.1. Influence of Interface Treatment

**Figure 10:**
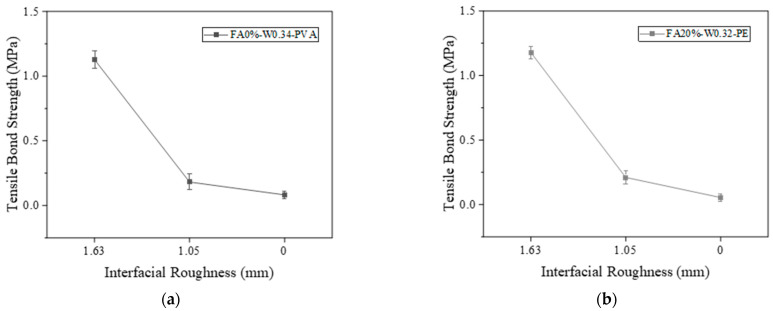
Effect of interface roughness on tensile bond strength: (a) FA0%-W0.34-PVA and (**b**) FA20%-W0.32-PE.

The bond interfaces include high-pressure water-jet-treated interface, chiseling interface, and smooth interface, with the interface roughness being 1.63 mm, 1.05 mm, and 0 mm, respectively. As shown in Figure 10a,b, the tensile bond strengths of PVA- and PE-fiber-reinforced samples decreased with the decrease in interface roughness. The reason may be that the larger the roughness of the bonding interface, the larger the contact area between EGC and concrete. Therefore, the more gel products produced by the chemical reaction between the geopolymer and the existing concrete, the greater the mechanical interlocking force, van der Waals force, and chemical cementation, and the higher the bond tensile strength of the sample [40].

**Figure 11:**
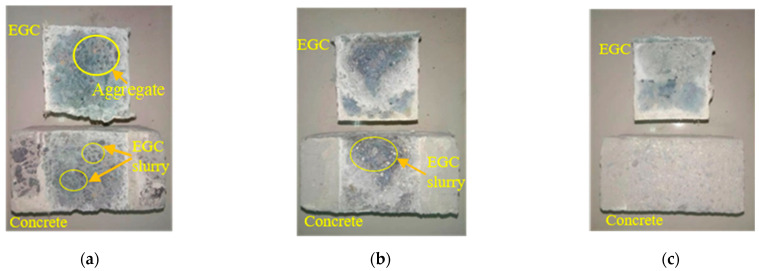
Tensile failure surface: (**a**) interface treated with high-pressure water jet, (**b**) chisel interface, and (**c**) smooth interface.

Figure 11 shows the tensile failure surfaces of different samples. From Figure 11a, it can be seen that dark green EGC slurry remains on the concrete when the interface is treated with the high-pressure water jet, and concrete aggregate remains on the surface of the EGC. Figure 11b shows that concrete grout remains on the EGC side at the chisel interface. Figure 11c shows that no slurry material remains on the concrete.

#### 3.3.2. Influence of FA Content

According to the previous exploration of mechanical properties, the FA contents of 0%, 20%, and 40% for PVA-fiber-reinforced EGC and the FA contents of 20%, 40%, and 60% for PE-fiber-reinforced EGC were selected to study the effect of FA content on the tensile bond strength of the interface treated with high-pressure water gun.

**Figure 12:**
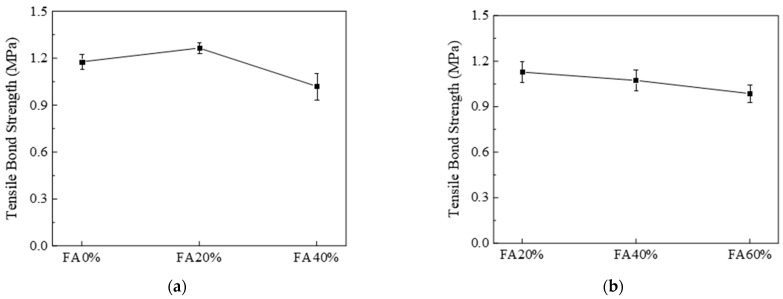
Effect of FA content on interfacial tensile bond strength; (**a**) W0.34-PVA and (**b**) W0.32-PE.

Figure 12 shows the tensile bond strength curves of PVA and PE-fiber-reinforced EGCs with different FA contents. As shown in Figure 12a, for the PVA-fiber-reinforced EGCs, the tensile bond strengths were 1.18 MPa, 1.26 MPa, and 1.02 MPa when the FA contents were 0%, 20%, and 40%, respectively. The tensile bond strengths of the samples with FA contents of 0% and 20% were higher than that of the sample with FA content of 40%. The reason for this phenomenon may be that C-S-H gel with low Ca/Si can be formed by the interaction between concrete and slag-based polymer at the interface for the sample with 0% FA, which is more dense than the C-S-H gel with high Ca/Si in the concrete and fills the voids at the interface [30]. For the samples with 20% FA, the appropriate amount of FA improves the fluidity of geopolymer slurry. When the slurry contacts with the surface of concrete, the hydration reaction is sufficient, and the reaction product is closely bonded with the concrete. For the sample with FA content of 40%, due to excessive FA content, the strength of the reaction product of the sample is low, which leads to the decrease in the compressive strength of the EGC matrix and thus the decrease in the tensile bond strength.

Figure 12b shows that the tensile bond strength of PE-fiber-reinforced EGC samples decreased with increasing FA content. When the FA contents were 20%, 40%, and 60%, the tensile bond strengths were 1.13 MPa, 1.07 MPa, and 0.99 MPa, respectively. The reason for this phenomenon may be that the activity of FA is low, and the dissociation and polycondensation process of the vitreous body is slow. In the process of alkali activation, Ca(OH)_2_ produced by the hydration reaction preferentially reacts with the active component after disaggregation of GGBS [30]. Therefore, for samples with excessive FA content, there is a large amount of unreacted FA, and the influence of unreacted FA affects the contact of alkali activation reaction products at the bonding interface with the existing concrete, leading to a decrease in bond strength.

#### 3.3.3. Influence of Water–Binder Ratio

**Figure 13:**
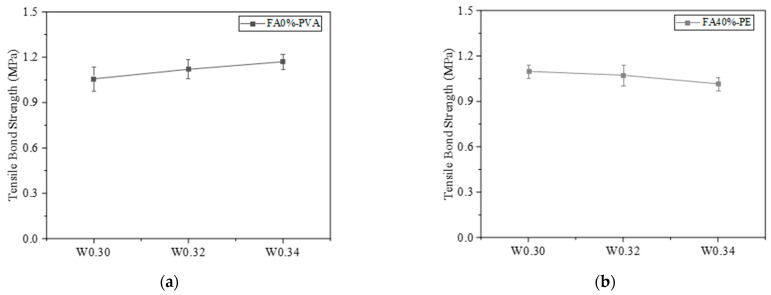
Effect of water–binder ratio on interfacial tensile bond strength: (**a**) FA0%-PVA and (**b**) FA40%-PE.

The FA0%-PVA and FA40%-PE samples were selected as the reference groups to explore the effect of water–binder ratio on interface bonding performance. Figure 13 shows the tensile bond strength curves of PVA- and PE-fiber-reinforced EGCs with various water–binder ratios. Figure 13a shows that, for PVA-fiber-reinforced EGCs, when the water–binder ratios were 0.30, 0.32, and 0.34, the tensile bond strengths were 1.05 MPa, 1.12 MPa, and 1.17 MPa, respectively. The tensile bond strength increased with the increase in water–binder ratio. Figure 13b shows that, for PE-fiber-reinforced EGCs, when the water–binder ratios were 0.30, 0.32, and 0.34, the tensile bond strengths were 1.10 MPa, 1.07 MPa, and 1.02 MPa, respectively. The tensile bond strength decreases with the increase in water–binder ratio. It can be seen from Figure 13 that the influence of water–binder ratio on the tensile bond strength for PVA and PE-fiber-reinforced EGCs is opposite. This is because, as discussed in Section 3.1.2, the hydrophilic functional groups on the surface of PVA fibers can absorb some of the water, which may lead to insufficient hydration reactions for PVA-reinforced EGCs with low water-binder-ratios (W0.30 and W0.32), and as the water–binder ratio increases, the hydration degree increases, increasing tensile bond strength [41]. For PE-reinforced EGCs, the hydration reaction is sufficient when the water–binder ratio is 0.30, and with the increase in water–binder ratio, the porosity at the interface increases, resulting in the decrease in tensile bond strength.

### 3.4. Single Shear Bond Performance

FA0%-W0.34-PVA and FA40%-W0.32-PE were selected as reference groups to explore the effects of interface treatment method, FA content, and water–binder ratio on ultimate shear strength due to their better mechanical properties.

#### 3.4.1. Influence of Interface Treatment

**Figure 14:**
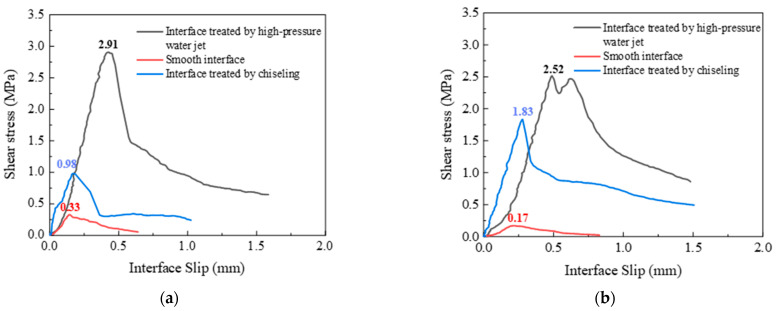
Effect of interface treatment on interface shear stress–interface slip: (**a**) FA0%-W0.34-PVA and (**b**) FA20%-W0.32-PE.

The bond interfaces include high-pressure water-jet-treated interface, chiseling interface, and smooth interface, with interface roughness of 1.63 mm, 1.05 mm and 0 mm, respectively. Figure 14 shows the shear stress–interface slip curves of PVA- and PE-fiber-reinforced EGCs with various interface treatments. As shown in Figure 14a, the ultimate shear strengths of PVA-fiber-reinforced EGCs are 2.91 MPa, 0.98 MPa, and 0.33 MPa, respectively. As shown in Figure 14b, the ultimate shear strengths of PE-fiber-reinforced EGCs are 2.52 MPa, 1.83 MPa, and 0.17 MPa, respectively. The results show that the interfacial roughness has a great influence on the ultimate shear strength. Mechanical interlocking force, van der Waals forces, and chemical cementation exist between the EGC and the concrete. The interface with the higher roughness has a larger contact area and is also subject to the stronger effects of the three forces, so the shear bond strength of the sample was higher.

**Figure 15:**
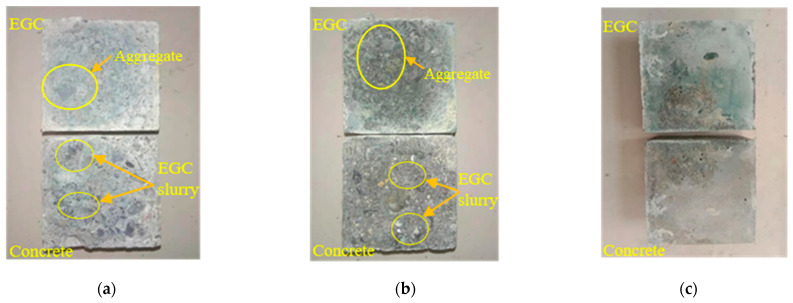
Shear failure face of samples: (**a**) interface treated with high-pressure water jet; (**b**) chiseling interface; and (**c**) smooth interface.

Figure 15 shows the shear failure face of different samples. For the samples with interfaces treated with high-pressure water jet and chiseling, the EGC slurry and fiber can be seen on the concrete side, and the concrete slurry and coarse aggregate adhere to the EGC side. For smooth interface samples, no residual EGC and concrete slurry were found on the surface.

#### 3.4.2. Influence of FA Content

**Figure 16:**
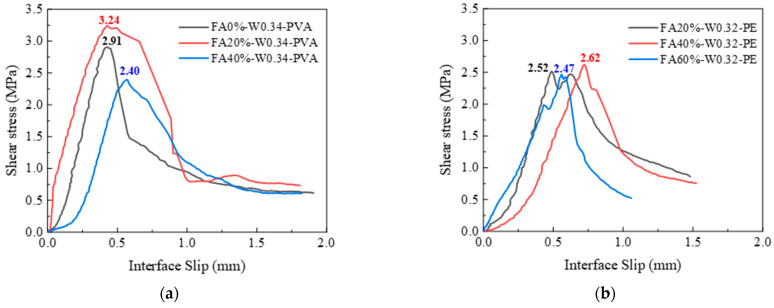
Effect of FA content on interface shear stress–interface slip: (**a**) W0.34-PVA and (**b**) W0.32-PE.

Figure 16 shows the shear stress–slip curve of PVA and PE-fiber-reinforced EGCs with different FA contents. Figure 15a shows that, for PVA-fiber-reinforced EGCs, when the FA contents were 0%, 20%, and 40%, the ultimate shear strengths were 2.91 MPa, 3.24 MPa, and 2.40 MPa, respectively. The results show that the ultimate shear strength of the sample with an FA content of 20% was better than those of the samples with FA contents of 0% and 40%. This is consistent with the tensile bond strength of PVA-reinforced EGCs in Section 3.3.2. Figure 16b shows that, for PE-fiber-reinforced EGCs, when the FA contents were 20%, 40%, and 60%, the ultimate shear strengths were 2.52 MPa, 2.62 MPa, and 2.47 MPa, respectively. Moreover, it can be seen from Figure 16b that, with the increase in FA content from 20% to 40%, the ultimate shear strength and corresponding slip value of the sample increased, but when the FA content increased to 60%, the ultimate shear strength and corresponding slip value decreased. The results show that, for PE-fiber-reinforced EGC, the samples with 40% FA had the best shear bond performance. This is because the sample with 40% FA content has good fluidity, which is conducive to the contact between the EGC slurry and the concrete. At the same time, it can be seen from Section 3.1.1 that the PE-fiber-reinforced EGC samples with 40% FA had sufficient hydration reaction and high compressive strength, resulting in high interfacial shear strength.

#### 3.4.3. Influence of Water–Binder Ratio

**Figure 17:**
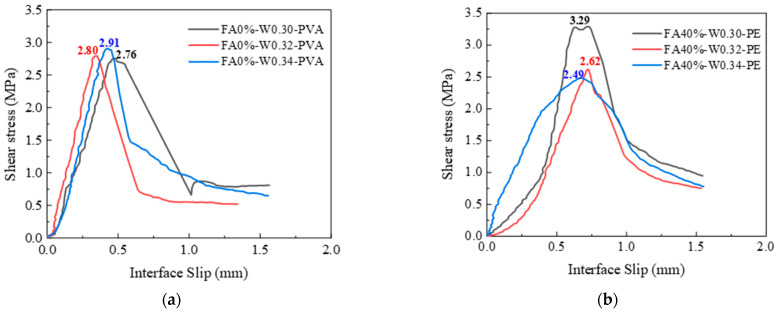
Effect of water–binder ratio on interface shear stress–interface slip: (**a**) FA0%-PVA and (**b**) FA40%-PE.

Figure 17 shows the shear stress–slip curves of PVA and PE-fiber-reinforced EGCs with different water–binder ratios. Figure 17a shows that the ultimate shear strengths of PVA-fiber-reinforced EGCs were 2.76 MPa, 2.80 MPa, and 2.91 MPa when the water–binder ratios were 0.30, 0.32, and 0.34, respectively. With the increase in water–binder ratio, the ultimate shear strengths of the samples increased slightly. The ultimate shear strength and corresponding ultimate slip value of the samples with a water–binder ratio of 0.34 were relatively large. The experimental results show that, for PVA-fiber-reinforced EGCs, the shear capacity of the interface was improved to a certain extent by increasing the water–binder ratio. This is consistent with the tensile bond strength of PVA-reinforced EGCs in Section 3.3.3.

Figure 17b shows that, for PE-fiber-reinforced EGCs, as the water–binder ratio increased from 0.30 to 0.34, the ultimate shear strength of the sample decreased gradually, and the ultimate shear strengths were 3.29 MPa, 2.62 MPa, and 2.49 MPa, respectively. The test results show that, for PE-fiber-reinforced EGCs, the ultimate shear strength decreased with the increase in water–binder ratio. This is consistent with the tensile bond strength of PE-reinforced EGCs in Section 3.3.3.

It can be seen from Figure 16 and Figure 17 that, before the samples reach the ultimate shear strength, the shear strength increased linearly with the increase in interface slip. When the samples reach the ultimate shear strength, in addition to the smooth interface, there is residual shear strength due to the mechanical interlocking between the EGC and the existing concrete and the bridging effect of the fiber at the interface. The reason for this phenomenon is that when the interfacial transition zone between EGCs and concrete is not destroyed under shear load, the shear bearing capacity is borne by chemical cementation, the van der Waals force, and the mechanical interlocking force. When the samples reach the ultimate shear strength, the chemical adhesive and van der Waals force gradually disappear, and the shear force is borne by the mechanical interlocking force and the bridging force of the fiber at the interface [39].

#### 3.4.4. Bond–Slip Model of EGC with Ordinary Concrete

In this study, a model of single shear bond strength considering water–binder ratio (*W*/*B*) and fly ash content (*F*) of EGC matrix is proposed.

Based on the test results from Section 3.4.2 and Section 3.4.3, this study considers the ultimate shear bond stress and its corresponding slip value and the interfacial fracture energy as eigenvalues, and proposes a bilinear bond–slip model, as shown in Equations (6)–(10) [42]:

(6) $$\left\{\begin{array}{l} \tau_{a}=\tau_{u}\frac{S}{S_{u}},0\leq S\leq S_{u}\\ \tau_{a}=\tau_{u}\frac{S_{f}-S_{u}}{S_{f}-S_{u}},S_{u}\leq S\leq S_{f}\\ \tau_{a}=0,S>S_{f} \end{array}\right.$$

where *τ_a_* is the shear bond stress (MPa); *τ_u_* is the ultimate shear bond stress (MPa); *S_u_* is the relative slip corresponding to the ultimate shear bond stress (mm); and *S_f_* is the maximum relative slip of the interface (mm), which can be calculated as follows:

(7) $$S_{f}=\frac{2G_{f}}{\tau_{u}}$$

where *G_f_* is the fracture energy (N/mm).

(8) $$\tau_{u}=\alpha \tau_{u1}\tau_{u2}$$

(9) $$S_{u}=\beta S_{u1}S_{u2}$$

(10) $$G_{u}=\gamma G_{u1}G_{u2}$$

where *α*, *β*, and *γ* are fitting coefficients; *τ_u_*_1_, *S_u_*_1_, and *G_f_*_1_ are influence coefficients of water–binder ratio (*W/B*) on *τ_u_*, *S_u_*, and *G_f_*, respectively; and *τ_u_*_2_, *S_u_*_2_, and *G_f_*_2_ are influence coefficients of fly ash content (*F*) on *τ_u_*, *S_u_*, and *G_f_*, respectively.

(1) PVA-fiber-reinforced EGC

**Figure 18:**
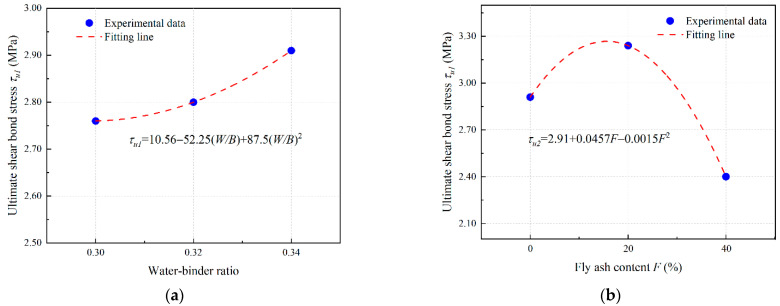
Determination of *τ_u_* of PVA fiber reinforced EGCs: curve fitting for (**a**) *τ_u_*_1_ and (**b**) *τ_u_*_2_.

**Figure 19:**
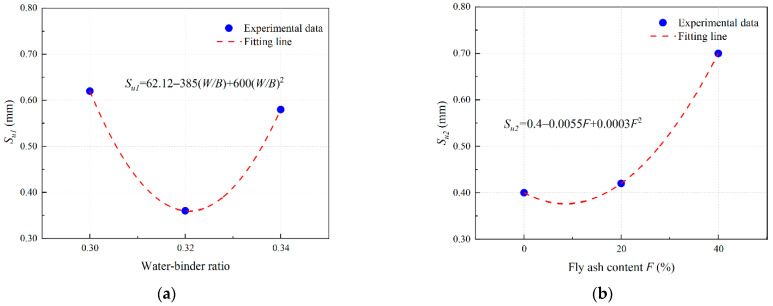
Determination of *S_u_* of PVA fiber reinforced EGCs: curve fitting for (**a**) *S_u_*_1_ and (**b**) *S_u_*_2_.

**Figure 20:**
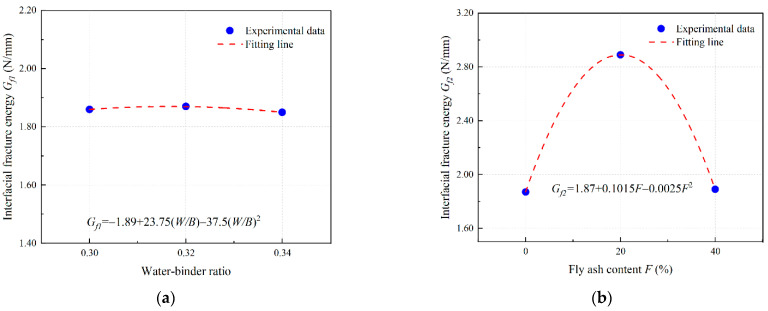
Determination of *G_f_* of PVA fiber reinforced EGCs: curve fitting for (**a**) *G_f_*_1_ and (**b**) *G_f_*_2_.

Based on the results of Section 3.4.2 and Section 3.4.3, the polynomial fitting results of the influence coefficient of PVA-fiber-reinforced EGC are obtained as shown in Figure 18, Figure 19 and Figure 20.

The fitting parameters *α* = 0.35, *β* = 2.05, and *γ* = 0.167 are obtained by nonlinear curve fitting. Equations (8)–(10) can be expressed in the form of Equations (11)–(13).

(11) $$\tau_{u}=0.351\left[2.91+0.0457F-0.0015F^{2}\right]$$

(12) $$S_{u}=2.05\left[62.12-385\left(W/B\right)+600{\left(W/B\right)}^{2}\right]\left[0.4-0.0055F+0.0003F^{2}\right]$$

(13) $$G_{f}=0.167\left[1.89+23.75\left(W/B\right)-37.5{\left(W/B\right)}^{2}\right]\left[1.87+0.1015F+0.0025F^{2}\right]$$

(2) PE-fiber-reinforced EGC

**Figure 21:**
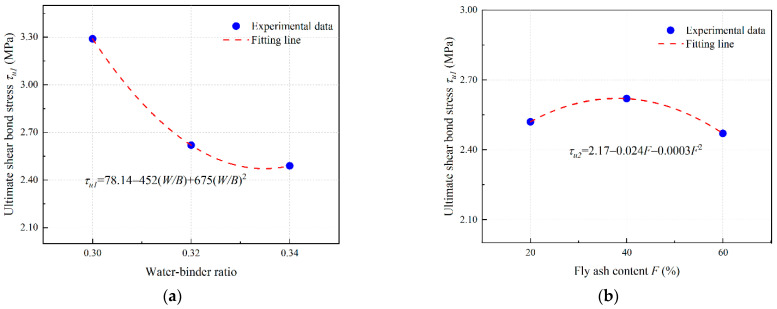
Determination of *τ_u_* of PE fiber reinforced EGCs: curve fitting for (**a**) *τ_u_*_1_ and (**b**) *τ_u_*_2_.

**Figure 22:**
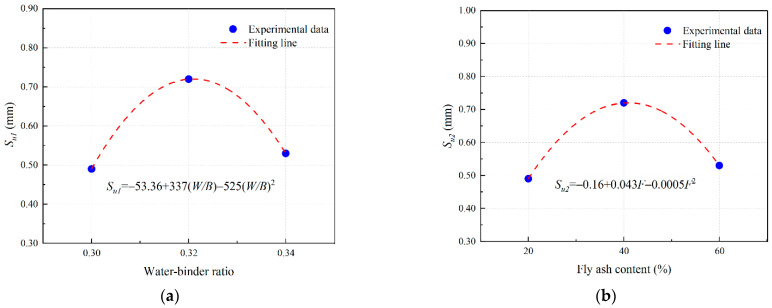
Determination of *S_u_* of PE fiber reinforced EGCs: curve fitting for (**a**) *S_u_*_1_ and (**b**) *S_u_*_2_.

**Figure 23:**
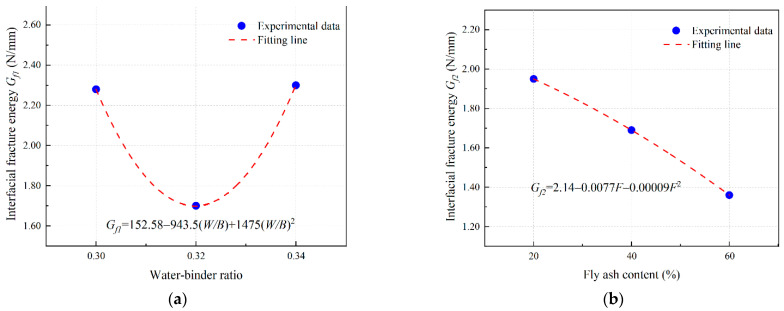
Determination of *G_f_* of PE fiber reinforced EGCs: curve fitting for (**a**) *G_f_*_1_ and (**b**) *G_f_*_2_.

Based on the results of Section 3.4.2 and Section 3.4.3, the polynomial fitting results of the influence coefficient of PE-fiber-reinforced EGC are obtained as shown in Figure 21, Figure 22 and Figure 23.

The fitting parameters *α* = 0.57, *β* = 1.3, and *γ* = 0.59 are obtained by nonlinear curve fitting. Equations (8)–(10) can be expressed in the form of Equations (14)–(16).

(14) $$\tau_{u}=0.57\left[78.14-452(W/B)+675{\left(W/B\right)}^{2}\right]\left[2.17-0.024F-0.0003F^{2}\right]$$

(15) $$S_{u}=1.3\left[-53.36+337\left(W/B\right)-525{\left(W/B\right)}^{2}\right]\left[-0.16+0.043F-0.0005F^{2}\right]$$

(16) $$G_{f}=0.591\left[152.58-943.5\left(W/B\right)+1475{\left(W/B\right)}^{2}\right]\left[2.14-0.0077F-0.0001F^{2}\right]$$

### 3.5. XRD Test

#### 3.5.1. Influence of FA Content

**Figure 24:**
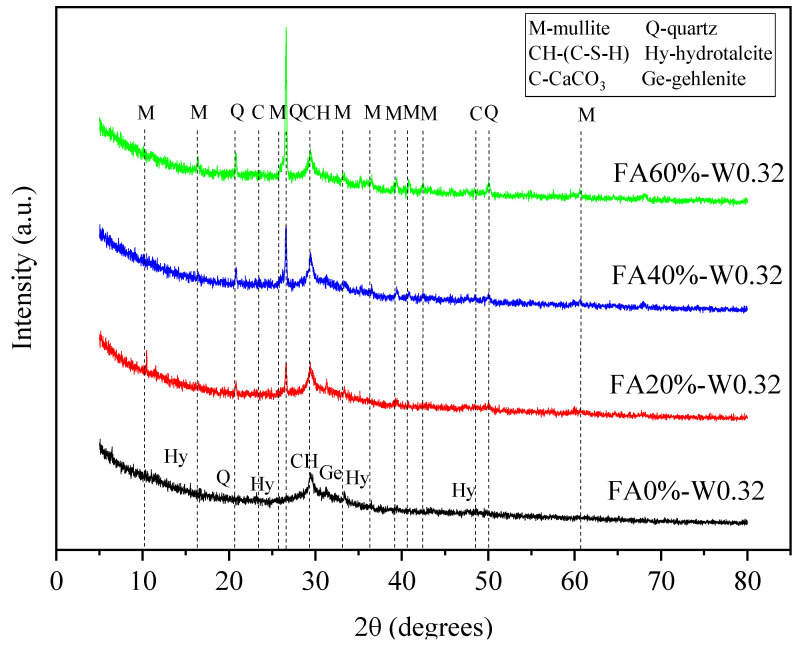
XRD spectra of geopolymers with different FA contents.

Figure 24 shows the XRD patterns of geopolymers with different FA contents. When the content of FA was 0%, the main reaction products of geopolymer were calcium silicate hydrate (C-S-H) (similar to riversideite, Ca_5_Si_6_O_16_(OH)_2_), abrazite (NaCa_2_Al_5_Si_5_O_20_∙6H_2_O), and hydrotalcite (Mg_6_Al_2_CO_3_(OH)_16_∙4H_2_O). With the addition of FA, mullite (Al_6_Si_2_O_13_) and hematite (Fe_2_O_3_) appeared, and their peak intensity increased with the increase in FA content. The peak intensity of C-S-H first increased and then decreased with the increase in FA content, in which 20% and 40% FA contents result in almost the same peak intensity. The results show that adding the proper amount of FA (FA20–40%) can enhance the reaction degree of geopolymer, but the addition of excessive FA (FA60%) can reduce the reaction degree.

#### 3.5.2. Influence of Water–Binder Ratio

**Figure 25:**
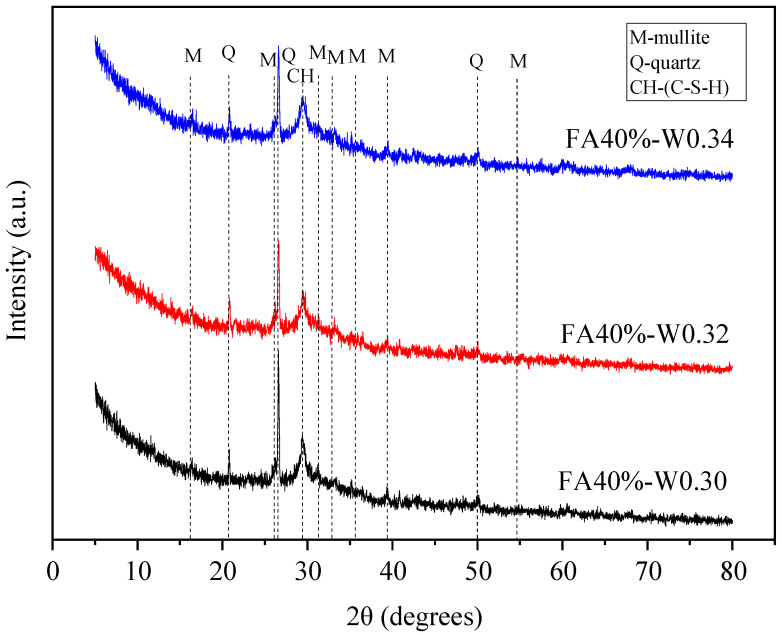
XRD spectra of geopolymers with different water–binder ratios.

Figure 25 shows the XRD patterns of geopolymer mixtures with different water–binder ratios. It can be seen that the peak intensity of the main reaction product C-S-H decreased with the increase in water–binder ratio. This is because the concentration of the alkali activator solution decreased with the increase in water content, resulting in the decrease in reaction degree and the decrease in C-S-H gel [43,44].

### 3.6. SEM Test

#### 3.6.1. Influence of FA Content

**Figure 26:**
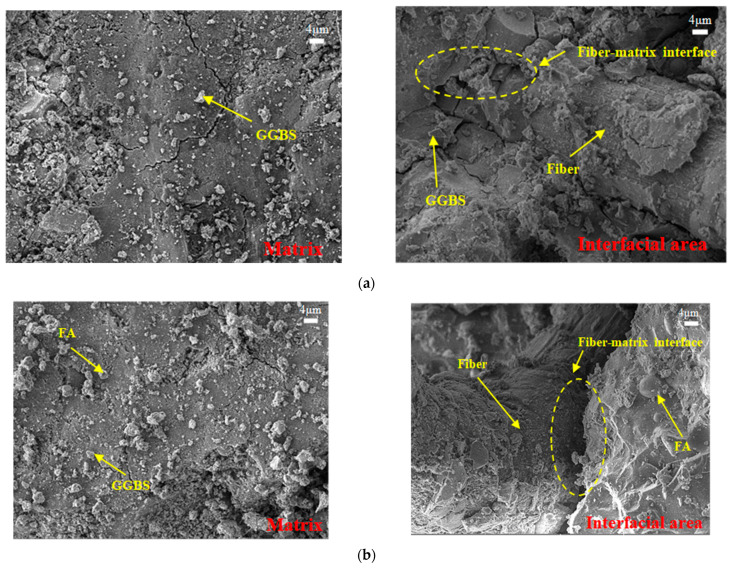
SEM images of the mixes FA0% and FA20% at 28 days: (**a**) FA0%-W0.32-PE and (**b**) FA20%-W0.32-PE.

The effect of FA on the microstructure of EGC was studied by analyzing the SEM images of FA0%-W0.32-PE and FA20%-W0.32-PE. Figure 26 shows the SEM images of FA0%-W0.32-PE and FA20%-W0.32-PE at 28 days. The left images in Figure 26a,b show that, for both mixtures, there are a large amounts of gel-like reaction products in both matrix and interface area. Meanwhile, a small amount of unreacted spherical FA particles and irregular wedge-shaped GGBS particles can be observed. Figure 26b shows that only a small amount of FA particles is observed in the interfacial area. Generally, FA particles can be easily recognized in this system due to their spherical shape and low reactivity [45]. This shows that, for FA20%-W0.32-PE, most of the FA particles have participated in the reaction.

Besides, it can be seen from Figure 26 that the two mixes have relatively dense microstructure. However, a close observation indicates that the void space of FA0%-W0.32-PE between the fiber and the matrix is less than that of FA20%-W0.32-PE, indicating a stronger fiber–matrix bonding. This difference may lead to different failure modes in uniaxial tensile tests. When the fiber–matrix interface strength is high, the fibers can be ruptured. However, if the fiber–matrix interface strength is low, the fibers tend to be pulled out [46]. The difference in the interface can explain why although the compressive strength of FA20%-W0.32-PE is similar to that of FA0%-W0.32-PE, the ultimate tensile strain is higher.

#### 3.6.2. Influence of Water–Binder Ratio

**Figure 27:**
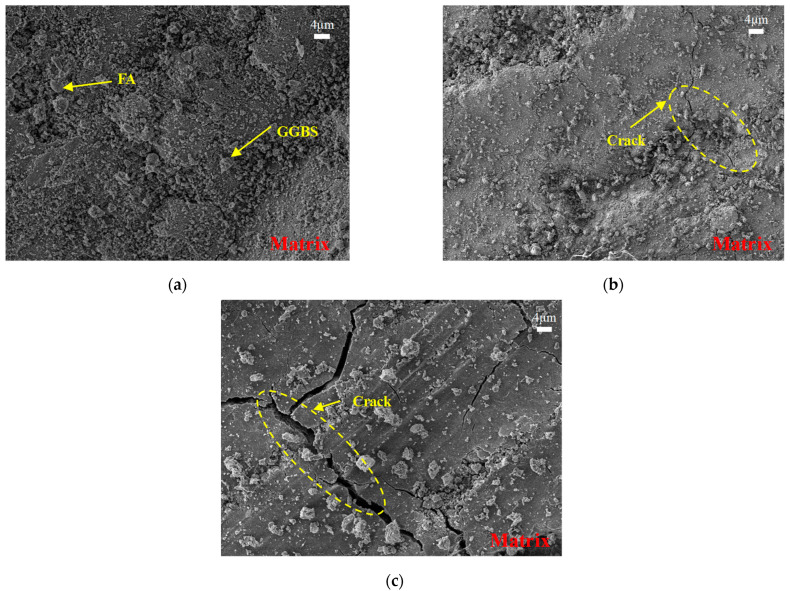
SEM images of the mixes (**a**) FA40%-W0.30-PE, (**b**) FA40%-W0.32-PE, and (**c**) FA40%-W0.34-PE at 28 days.

The effect of water–binder ratio on the microstructure of PE-fiber-reinforced EGC was studied by analyzing the SEM images of FA40%-W0.30-PE, FA40%-W0.32-PE, and FA40%-W0.34-PE at 28 days, as shown in Figure 27. It can be seen from Figure 27 that, with the increase in the water–binder ratio, the cracks in the EGC matrix gradually increased, and the compactness of the matrix gradually became worse. Therefore, PE-fiber-reinforced EGCs with a low water–binder ratio have a higher compressive strength.

## 4. Conclusions

In this study, the mechanical properties (compressive and uniaxial tensile properties), microstructure characteristics, and interfacial bonding properties of PVA and PE-fiber-reinforced EGCs were studied. The influences of FA content and water–binder ratio on the mechanical properties and the effects of interface treatment, FA content, and water–binder ratio on the bonding properties were discussed. The following conclusions can be drawn:

1. The strength and ductility of PE-fiber-reinforced EGCs can be improved by adding the proper amount of FA, while excessive FA can lead to a decrease in strength. For PE-fiber-reinforced EGCs with 20% FA, compressive strength, ultimate tensile stress, and ultimate tensile strain at 28 days can reach 79.1 MPa, 5.41 MPa, and 9.3%, respectively. Compared to PE-fiber-reinforced EGCs, the overall tensile properties of PVA-fiber-reinforced EGCs are worse. However, the interfacial bond strength between the two types of fiber-reinforced EGC and existing concrete is similar. This indicates that a higher ductility does not lead to higher bond strength.
2. For PVA-fiber-reinforced EGCs, the tensile bond strength and ultimate shear strength increase with a increase in FA content (0–40%), and the sample with 20% FA had the best bonding properties. However, with the change in FA content (20–60%), the tensile bond strength and ultimate shear strength of PE-fiber-reinforced EGCs showed little change, and the sample with 40% FA had the best bonding properties.
3. The bonding properties of PVA- and PE-fiber-reinforced EGCs have different trends with the increase in water–binder ratio. This may be because PE fiber is hydrophobic whereas PVA fiber is hydrophilic. However, the trends in compressive strength and interfacial bond strength with the increase in water–binder ratio are consistent, which indicates that there is a certain correlation between compressive strength and interfacial bond strength.
4. The interfacial bond strength of PVA and PE-fiber-reinforced EGCs decreased with the decrease in interface roughness. The size of effects of different interface treatments on the bonding performances of PVA and PE-fiber-reinforced EGCs in descending order was high-pressure water-jet-treated interface > chiseling interface > smooth interface.
5. The XRD studies showed that, when the FA content is 20–40%, the content of C-S-H gels is high and the reaction is sufficient. Additionally, the content of C-S-H gels decreases as the water-to-binder ratio increases from 0.30 to 0.34. That is, as the water-to-binder ratio increases, the concentration of the alkali activator solution decreases, resulting in a decrease in the degree of geopolymer reaction.
6. The SEM studies showed that a dense microstructure is formed in PE-fiber-reinforced EGCs. When the FA content is 20%, the fiber–matrix bonding is weakened, leading to the improvement of ductility. Additionally, with the increase in the water–binder ratio (0.30–0.34), the reaction products of the PE-fiber-reinforced EGC matrix gradually decrease, and the compactness of the matrix gradually deteriorates. This may be the reason why the interfacial bond strength between EGC and existing concrete decreases with the increase in water–binder ratio.

## Figures and Tables

**Table 1:** Chemical compositions of FA and GGBS (wt.%).

	SiO_2_	Al_2_O_3_	CaO	Fe_2_O_3_	Na_2_O	MgO
FA	53.6	29.7	3.4	5.9	1.2	1.3
GGBS	32.1	17	35.3	0.3	0.59	9.9

Note: All values in mass %.

**Table 2:** Properties of PVA and PE fibers.

Type of Fiber	Diameter (µm)	Length (mm)	Tensile Strength (MPa)	Elongation (%)	Elastic Modulus (GPa)	Density (g/cm^3^)
PVA	40	12	1560	6.5	41	1.3
PE	24	12	3000	2–3	110	0.98

**Table 3:** Mix proportions.

Mix ID	Binder		Activator	Water	Fiber Type	Fiber Content (vol.%)
	FA	GGBS				
FA0%-W0.30-PVA	0	1	0.04	0.30	PVA	2
FA0%-W0.32-PVA	0	1	0.04	0.32		2
FA0%-W0.34-PVA	0	1	0.04	0.34		2
FA20%-W0.32-PVA	0.2	0.8	0.04	0.32		2
FA40%-W0.32-PVA	0.4	0.6	0.04	0.32		2
FA60%-W0.32-PVA	0.6	0.4	0.04	0.32		2
FA0%-W0.32-PE	0	1	0.04	0.32	PE	2
FA20%-W0.32-PE	0.2	0.8	0.04	0.32		2
FA40%-W0.30-PE	0.4	0.6	0.04	0.30		2
FA40%-W0.32-PE	0.4	0.6	0.04	0.32		2
FA40%-W0.34-PE	0.4	0.6	0.04	0.34		2
FA60%-W0.32-PE	0.6	0.4	0.04	0.32		2

Note: Except for fiber content (volume fraction), all numbers are mass ratios.

## Data Availability

The data presented in this study are available on request from the corresponding author. The data are not publicly available due to privacy.
